# Copper Deficiency and Other Micronutrient Deficiencies During Prolonged Jejunal Versus Gastric Enteral Nutrition in Children

**DOI:** 10.3390/nu18213460

**Published:** 2026-10-22

**Authors:** Judith Raya-Muñoz, Blanca Maria del Castillo Martinez, Marta Freixas Bermejo, Marina Álvarez-Beltrán, Vanessa Cabello-Ruiz, Susana Redecillas-Ferreiro, Oscar Segarra-Cantón

**Affiliations:** Pediatric Gastroenterology and Clinical Nutrition Unit, Department of Pediatrics, Hospital Universitari Vall d’Hebron, Vall d’Hebron Barcelona Hospital Campus, Passeig de la Vall d’Hebron 119–129, 08035 Barcelona, Spain

**Keywords:** copper deficiency, enteral nutrition, jejunal feeding, post-pyloric nutrition, paediatrics, micronutrients, gastrostomy, trace elements

## Abstract

**Background/Objectives**: Copper deficiency is described in adults on prolonged jejunal enteral nutrition, but paediatric evidence remains scarce. We compared the prevalence, timing and consequences of copper and other micronutrient deficiencies in children on prolonged jejunal versus gastric enteral nutrition. **Methods**: Ambispective observational cohort study at a tertiary paediatric centre. Children aged 0–18 years receiving at least 80% of their nutritional requirements through an enteral device for more than three months were studied; pre-existing malabsorption was an exclusion criterion. Groups were defined by feeding route (jejunal or gastric). Serum copper, zinc, iron and selenium were assessed longitudinally, with concurrent *C*-reactive protein < 2 mg/dL. **Results**: Forty-six patients were included (jejunal, 31; gastric, 15). Copper deficiency occurred in 9 of 31 jejunal patients (29.0%) and in no gastric patient (risk difference 29.0%, 95% CI 4.9–46.6%; *p* = 0.021). Iron deficiency was the commonest abnormality (71% versus 60%; *p* = 0.51). Six of the nine copper-deficient children developed anaemia or neutropenia; all normalised after oral copper sulfate given through the gastric route. **Conclusions**: In this single-centre cohort, prolonged jejunal feeding was associated with more frequent copper deficiency despite adequate intake; larger studies should confirm this. We suggest monitoring serum copper from three months after post-pyloric feeding starts.

## 1. Introduction

Long-term enteral nutrition (EN) is an essential component of care for children with chronic dysphagia, neurological impairment, congenital malformations, and complex gastrointestinal disorders. As endoscopic and radiological techniques have evolved, gastrostomy and post-pyloric enteral access have become increasingly available, enabling sustained nutritional support that minimises hospitalisation and improves quality of life. The indications, access techniques and follow-up of long-term gastric and post-pyloric feeding in children are addressed by ESPGHAN position papers and guidelines [1,2,3] and by ESPEN and ASPEN guidance on home enteral nutrition and safe enteral practice [4,5]. Post-pyloric feeding is generally reserved for children who do not tolerate gastric feeding or in whom it is unsafe, most commonly because of severe gastro-oesophageal reflux disease (GERD) with recurrent aspiration, gastroparesis or other gastric dysmotility, or gastric outlet obstruction [1,6].

Although commercial enteral formulas are designed to meet macro- and micronutrient recommendations, deficiencies may still develop during prolonged feeding, particularly when physiological absorption mechanisms are bypassed. Micronutrient deficiencies are frequently underdiagnosed because their clinical manifestations are nonspecific and may be attributed to underlying disease. Standard paediatric enteral formulas marketed in the European Union are regulated as food for special medical purposes and, when nutritionally complete, must provide between 60 and 500 μg of copper per 100 kcal [7]; when prescribed to cover energy requirements they therefore usually meet or exceed reference copper intakes [8,9].

Copper is an essential trace element that participates in mitochondrial respiratory chain function, antioxidant defence, iron metabolism and mobilisation, haematopoiesis, connective tissue synthesis, and neurological integrity. Its absorption occurs principally in the stomach, duodenum, and proximal jejunum. Gastric acidity facilitates solubilisation of dietary copper, which is a prerequisite for efficient uptake via the copper transporter CTR1 in the intestinal epithelium [10,11]. When feeding bypasses the stomach and proximal small bowel, this early-phase absorption is lost, potentially compromising copper bioavailability despite adequate intake.

Copper deficiency manifests as refractory anaemia, neutropenia, pancytopenia, and bone marrow dysplasia, and in more severe or chronic presentations as sensory ataxia, peripheral neuropathy, and myelopathy [12,13]. In paediatric patients, these findings may closely mimic myelodysplastic syndromes or haematological malignancies, leading to extensive and unnecessary diagnostic workups before the correct diagnosis is established.

Nishiwaki et al. reported significantly lower serum copper concentrations and a higher prevalence of clinically relevant copper deficiency among adult patients receiving prolonged percutaneous endoscopic jejunostomy (PEJ) feeding compared with those receiving gastrostomy feeding [14]. However, comparable evidence in the paediatric population is lacking, with available data limited to isolated case reports and small case series describing refractory cytopenias in children fed through jejunal or gastrojejunal tubes [15,16,17], a concern also raised by others [18]. Acquired copper deficiency has likewise been reported after surgical exclusion of the duodenum and proximal jejunum, as in Roux-en-Y gastric bypass [19], and in children with intestinal failure [20].

The primary aim of this study was to evaluate and compare the prevalence of copper deficiency in children receiving prolonged jejunal versus gastric enteral nutrition. Secondary objectives included the assessment of iron, zinc, and selenium deficiencies; the determination of time to deficiency onset; the characterisation of clinical manifestations; and the evaluation of the response to copper supplementation.

## 2. Materials and Methods

### 2.1. Ethics Statement

This observational study was conducted at a tertiary university referral centre. The study protocol was reviewed and approved by the Clinical Research Ethics Committee (Comitè d’Ètica d’Investigació amb medicaments, CEIm) of Hospital Universitari Vall d’Hebron (protocol code PR(AMI)013/2025; date of approval 10 March 2025), and the study was conducted in accordance with the principles of the Declaration of Helsinki and with Spanish Organic Law 3/2018 on the protection of personal data. Written informed consent was obtained from parents or legal guardians, and assent was obtained from patients aged ≥12 years where applicable.

### 2.2. Study Design and Participants

Children and adolescents aged 0–18 years (one patient aged 21 years was included due to ongoing paediatric follow-up; a sensitivity analysis excluding this patient is reported in Section 3.2) receiving long-term EN between 2023 and 2025 were enrolled. The study followed an ambispective design and the cohort comprised two subsets: (i) children in whom the enteral device was placed during the recruitment period, who were enrolled at the time of device placement and in whom all determinations, including the baseline panel obtained before or at initiation of feeding, were collected prospectively (*n* = 17; JN, *n* = 15; GN, *n* = 2); and (ii) children already receiving EN when recruitment began, who were enrolled prospectively and followed prospectively thereafter, but whose baseline status and determinations performed between initiation of EN and enrolment were retrieved from the electronic clinical record (*n* = 29; JN, *n* = 16; GN, *n* = 13). All participants were followed prospectively from enrolment to the end of the study period. Inclusion criteria were: (1) receipt of ≥80% of nutritional requirements through an enteral feeding device; (2) duration of EN > 3 months; and (3) availability of serial micronutrient assessments during follow-up. Patients with a known malabsorptive disorder established before initiation of EN were excluded. For this purpose, a malabsorptive disorder was defined as intestinal failure requiring parenteral nutrition, cystic fibrosis or other exocrine pancreatic insufficiency, coeliac disease or inflammatory bowel disease. Children with anatomical abnormalities of the gastrointestinal tract who were fully enterally fed and did not meet this definition were not excluded; the one such patient with short bowel anatomy is described in Section 3.1, and a sensitivity analysis excluding her is reported in Section 3.2.

The selection of the study population and its allocation to the study groups are summarised in Figure 1, in accordance with the STROBE statement for observational studies [21].

Participants were classified according to feeding route: the jejunal nutrition group (JN) comprised patients receiving nutrition via transpyloric tube or gastrojejunostomy; the gastric nutrition group (GN) comprised patients receiving nutrition via nasogastric tube or gastrostomy. The feeding route was selected by the treating team according to the clinical indication, in line with ESPGHAN recommendations [1,2]: gastric access was the default, and post-pyloric feeding was used when gastric feeding was not tolerated or was considered unsafe. The specific indications in each group are detailed in Table 1.

Time zero was defined as the date on which the current feeding route was established. For every patient the first micronutrient panel obtained after initiation of EN was taken as the baseline value. The onset of a deficiency was defined as the date of the first interpretable determination falling below the lower reference limit, and the interval to deficiency was computed from time zero to that date. This interval could be documented in 7 of the 9 children who developed copper deficiency; in the remaining 2 the enteral device had been placed before the recruitment period and no determination preceding the first deficient value was retrievable, so these patients contribute to the prevalence estimate but not to the time-to-deficiency analysis. This distinction is addressed further in the Limitations section.

### 2.3. Data Collection

For each patient, the following variables were recorded: age, sex, underlying diagnosis, type of enteral access device, duration of EN, enteral formula utilised, and estimated daily micronutrient intake. Micronutrient intake was calculated based on the manufacturer’s declared nutritional composition and actual daily formula volume administered. Daily copper intake was expressed in μg/day and μg/kg/day and as a percentage of the age-specific recommended dietary allowance (RDA) of the US Institute of Medicine (IOM) [8] and of the adequate intake (AI) set by the European Food Safety Authority (EFSA) [9]; when the formula or its volume changed during follow-up, the intake recorded closest to each micronutrient determination was used. The brand and type of formula (polymeric, peptide-based or amino acid-based), previous gastrointestinal surgery, short bowel anatomy and the use of acid-suppressive medication were also recorded as potential determinants of micronutrient status. In addition, ceruloplasmin, full blood count (haemoglobin, mean corpuscular volume, neutrophil and platelet counts), reticulocyte count, serum ferritin, transferrin saturation and serum albumin were recorded at the assessments performed in patients under investigation for a suspected deficiency. *C*-reactive protein (CRP) was measured in the same blood sample as every micronutrient determination, because copper and ceruloplasmin are positive acute-phase reactants whose serum concentrations rise with systemic inflammation independently of nutritional status, whereas zinc behaves as a negative acute-phase reactant. Following the ESPEN micronutrient guideline, a determination was considered interpretable only when the concurrent CRP was below 2 mg/dL [22]; determinations obtained during an intercurrent infection or with a CRP at or above that threshold were disregarded and repeated after clinical resolution.

### 2.4. Micronutrient Assessment

Serial serum concentrations of copper, iron, zinc, and selenium were obtained during routine outpatient follow-up. Reference intervals applied were: copper 70–150 μg/dL; iron 65–175 μg/dL; zinc 45–110 μg/dL; selenium 60–120 μg/L; ceruloplasmin 20–60 mg/dL [22]. Only determinations obtained with a concurrent CRP below 2 mg/dL were used to classify micronutrient status. Micronutrient levels below these thresholds on at least one determination were defined as deficiency. For iron, deficiency was defined operationally as a serum iron concentration below 65 μg/dL on at least one interpretable determination. Because serum iron is subject to diurnal variation and does not reflect body iron stores, serum ferritin and transferrin saturation were additionally reviewed when available, and a ferritin concentration below 12 μg/L in children younger than 5 years or below 15 μg/L in older children, or a transferrin saturation below 16%, was considered to confirm depleted iron stores [23]. For patients with copper deficiency, ceruloplasmin concentration, full blood count, reticulocyte count, and clinical symptomatology were additionally reviewed. Ceruloplasmin, the principal copper-transport protein and an indirect marker of functional copper status, was used to support the diagnosis in equivocal cases.

### 2.5. Outcomes

The primary outcome was the prevalence of copper deficiency, defined as at least one serum copper determination below the lower reference limit. Secondary outcomes included: (1) prevalence of iron, zinc, and selenium deficiencies; (2) time from initiation of EN to first deficient determination; (3) coexistence of multiple deficiencies; (4) clinical manifestations attributable to copper deficiency; (5) requirement for supplementation; and (6) biochemical and haematological response to treatment.

### 2.6. Statistical Analysis

Continuous variables are expressed as median and range or mean ± standard deviation according to data distribution as assessed by the Shapiro–Wilk test. Categorical variables are presented as frequencies and percentages. Between-group comparisons for categorical variables were performed using Fisher’s exact test; continuous variables were compared using the Mann–Whitney U test. The between-group difference in the prevalence of each deficiency was expressed as a risk difference with its 95% CI, calculated with the Newcombe hybrid score method [24]; odds ratios were not estimated because no copper, zinc or selenium deficiency occurred in the GN group. Because micronutrients were measured at unequal intervals during routine follow-up, the true onset of a deficiency is interval-censored, lying between the last normal and the first deficient determination. For the time-to-event analysis, onset was assigned to the date of the first deficient determination, patients who did not develop deficiency were censored at their last interpretable determination, and the two copper-deficient patients without any determination preceding the first deficient value were excluded. Time to deficiency was described with Kaplan–Meier curves and compared with the log-rank test; given the interval-censored data, the resulting times represent upper-bound estimates and are interpreted descriptively. To account for repeated measurements, serum concentrations were compared using a single summary value per patient (the median of each patient’s interpretable determinations), so that patients sampled more frequently were not over-represented. Two sensitivity analyses of the primary outcome were performed: (i) excluding the patient aged 21 years and (ii) excluding the patient with short bowel anatomy; both exclusions were also combined. Multivariable adjustment for potential confounders was not feasible because of the small number of events and the absence of events in the GN group. A two-tailed *p* value < 0.05 was considered statistically significant. Statistical analyses were performed using SPSS Statistics v26 (IBM Corp., Armonk, NY, USA).

## 3. Results

### 3.1. Patient Characteristics

A total of 46 patients were included: 31 in the JN group and 15 in the GN group. Demographic and clinical characteristics are summarised in Table 1. The majority of patients in both groups were male and younger than 10 years of age. Dysphagia with reflux was the most frequent indication for enteral access, accounting for 54.8% of JN patients. The specific clinical indications for jejunal rather than gastric access, together with the type of device, underlying diagnoses, gastrointestinal surgical history, formula type, duration of EN, number of determinations and actual copper intake, are detailed in Table 1. The most common underlying diagnoses included neurological impairment, structural gastrointestinal anomalies, congenital syndromes, and complex chronic diseases. All patients received standard, nutritionally complete commercial paediatric enteral formulas, whose brand names, composition and copper content are shown in Table 2. Formula copper content ranged from 75 to 120 μg/100 mL (median 80 μg/100 mL) in both groups, equivalent to 72–100 μg/100 kcal. The actual median daily copper intake was 864 μg/day (46.5 μg/kg/day) in the JN group and 888 μg/day (49.3 μg/kg/day) in the GN group (*p* = 0.51), corresponding to a median of 171% and 200% of the age-specific IOM RDA and 86% and 96% of the EFSA AI, respectively. In the nine children who developed copper deficiency, copper intake at the time of diagnosis ranged from 107% to 249% of the RDA (59–105% of the EFSA AI) and did not differ from that of the JN patients who remained copper-sufficient (median 172% vs. 170% of the RDA; *p* = 0.88). One JN patient (patient 1 in Section 3.5) had short bowel anatomy secondary to neonatal resection of a type IIIb jejunal atresia, with a residual small-bowel length of 85 cm and a preserved ileocaecal valve; she had been weaned from parenteral nutrition and fully enterally fed for 14 months before enrolment, and did not meet the predefined definition of a malabsorptive disorder.

### 3.2. Prevalence of Micronutrient Deficiencies

The prevalence of micronutrient deficiencies across both groups is shown in Table 3. Copper deficiency was detected in 9 of 31 JN patients (29.0%) and in none of the 15 GN patients (risk difference 29.0%, 95% CI 4.9–46.6%; *p* = 0.021, Fisher’s exact test). Iron deficiency was the most frequent abnormality overall, occurring in 22 JN patients (71.0%) and 9 GN patients (60.0%; *p* = 0.51). When the stricter criteria based on ferritin or transferrin saturation were applied, depleted iron stores were confirmed in 14 of the 22 JN patients (63.6%) and 6 of the 9 GN patients (66.7%) with low serum iron; in the remaining patients ferritin and transferrin saturation were normal (JN, *n* = 5; GN, *n* = 2) or not available (JN, *n* = 3; GN, *n* = 1). Selenium deficiency was identified in four JN patients (12.9%) and none in the GN group (*p* = 0.29). Zinc deficiency was detected in one JN patient (3.2%), whose value was borderline (40 μg/dL) and normalised spontaneously on the following determination; the four selenium values were likewise borderline (45–50 μg/L) and all normalised without intervention, so that neither the zinc nor the selenium deficits required supplementation. Only one patient, belonging to the JN group, presented a combined deficiency of copper, iron, and selenium. Within the GN group, iron deficiency was the only micronutrient abnormality detected; no patient in this group developed copper, zinc or selenium deficiency.

Because no copper deficiency occurred in the GN group, the upper limit of the 95% CI for its prevalence in that group was 20.4%, and the lower limit of the CI for the risk difference approached zero; the size of the excess risk is therefore estimated imprecisely. In the sensitivity analyses, exclusion of the patient aged 21 years, who belonged to the GN group and did not develop copper deficiency, did not materially change the result (9/31 [29.0%] vs. 0/14; risk difference 29.0%, 95% CI 3.9–46.6%; *p* = 0.040). Exclusion of patient 1, who had short bowel anatomy, yielded a similar estimate (8/30 [26.7%] vs. 0/15; risk difference 26.7%, 95% CI 2.8–44.4%; *p* = 0.038), as did exclusion of both patients (8/30 [26.7%] vs. 0/14; risk difference 26.7%, 95% CI 1.8–44.4%; *p* = 0.041).

### 3.3. Serum Concentrations and Inflammatory Status

Serum concentrations of the parameters recorded during follow-up are summarised in Table 4. Median serum copper was lower in the JN group than in the GN group (89.2 vs. 98.9 μg/dL; *p* = 0.03), whereas no significant between-group differences were observed for iron, zinc or selenium. Ceruloplasmin paralleled serum copper (22.5 vs. 25.8 mg/dL; *p* = 0.02). Every determination included in the analysis was obtained with a concurrent CRP below 2 mg/dL, the threshold above which serum copper and ceruloplasmin cannot be interpreted as nutritional markers; no copper measurement was therefore made in the context of ongoing systemic inflammation. This is relevant to the interpretation of our findings, because an acute-phase response raises rather than lowers serum copper and would consequently have led to underestimation, not overestimation, of the prevalence of deficiency.

### 3.4. Timing of Micronutrient Deficiencies

Micronutrient deficiencies were detected earlier in the JN group than in the GN group. Among JN patients, copper deficiency was first detected at a median of 170 days (range 75–450 days) after initiation of jejunal feeding, calculated over the 7 of 9 patients with a documented interval, with the majority of cases concentrated within the first six months. In the Kaplan–Meier analysis, the cumulative probability of copper deficiency in the JN group was 14.3% at 6 months and 22.7% at 12 months, versus 0% in the GN group (log-rank *p* = 0.035). Because the true onset lies between the last normal and the first deficient determination, and because the interval between consecutive determinations varied (median 122 days, range 55–224; more frequent in the JN group, Table 1), these times are upper-bound estimates conditioned by the sampling schedule and should be interpreted with caution. Iron deficiency appeared at a median of 194 days in the JN group versus 264 days in the GN group. Selenium deficiency was detected only in JN patients (4 of 31, 12.9%) and zinc deficiency in a single JN patient (3.2%). Because of these small numbers, the borderline and transient nature of the values, and the unequal spacing of determinations, time to deficiency was not formally estimated for selenium or zinc, and the corresponding cells are reported as not applicable in Table 3.

### 3.5. Copper Deficiency: Clinical Characteristics and Management

Clinical and biochemical details of all nine patients with copper deficiency are presented in Table 5. Six patients (66.7%) developed clinical manifestations: five presented anaemia (including one case with severe anaemia and bone marrow dysplasia with ring sideroblasts) and one patient developed isolated neutropenia. Three patients remained asymptomatic and were identified exclusively through routine biochemical surveillance. At the time copper deficiency was identified, CRP was below 2 mg/dL in all nine patients, excluding an acute-phase response as an explanation for the biochemical findings; ceruloplasmin was concordantly reduced in 7 of the nine patients. Individual values are detailed in Table 6. The index case illustrates the presentation: a 2-year-old girl with Down syndrome, repaired type 1 oesophageal atresia and short bowel syndrome fed through a gastrojejunostomy developed progressive anaemia (haemoglobin nadir 6 g/dL) with a blocked iron profile (transferrin saturation 21%), normal vitamin B12 and folate, and subsequent neutropenia unresponsive to intravenous iron; bone marrow aspiration showed trilineage dysplasia with ring sideroblasts before serum copper was found to be 6.9 μg/dL with a low ceruloplasmin, normal zinc and a negative CRP.

All six symptomatic patients received copper sulfate supplementation administered through the gastric route at doses of 40–50 μg/kg/day. In the index case, copper was initially replaced intravenously (as part of a trace-element preparation delivering 170 μg of copper daily) because of diarrhoea and faltering growth, and was then switched to oral copper sulfate 250 μg every 12 h administered through the gastric port; serum copper had risen to 21 μg/dL at one month and was normal at two months. Copper concentrations normalised in all treated patients, and associated haematological abnormalities progressively resolved within two months of treatment initiation. Three patients with mildly reduced copper levels without clinical manifestations did not require treatment: two achieved spontaneous normalisation during follow-up and one transitioned to gastric feeding, which was followed by recovery of copper levels.

## 4. Discussion

In this ambispective cohort study, prolonged jejunal enteral nutrition was associated with a higher prevalence of copper deficiency in paediatric patients compared with gastric feeding (29.0% vs. 0%; risk difference 29.0%, 95% CI 4.9–46.6%), with deficiency developing despite formula provision that met recommended daily copper intakes. To our knowledge, this is one of the largest paediatric cohorts specifically comparing micronutrient status according to enteral feeding route, and one of the few to describe the time to deficiency onset in this population. The wide confidence interval, the small number of events and the observational design mean, however, that the magnitude of this association and its independence from the underlying disease must be interpreted with caution.

Our findings are consistent with evidence from adult studies. Nishiwaki et al. reported significantly lower serum copper concentrations from six months onwards in adult patients receiving percutaneous endoscopic jejunostomy (PEJ) compared with percutaneous endoscopic gastrostomy (PEG) feeding, with severe copper deficiency accompanied by neutropenia and anaemia in six of 23 PEJ patients [14]. Our paediatric results extend these observations, suggesting that route-dependent impairment of copper absorption is not limited to the adult population and occurs even in the context of heterogeneous underlying diagnoses and paediatric physiology [25]. The paediatric case reports and small series published to date describe a consistent picture of refractory anaemia and neutropenia, sometimes with dysplastic bone marrow changes, in children fed exclusively through jejunal or gastrojejunal tubes with formulas whose copper content met recommendations, followed by prompt haematological recovery after copper replacement [15,16,17]. Haematological disorders caused by copper deficiency have also been described in adult tube-fed patients [26,27,28] and after bariatric procedures that bypass the duodenum and proximal jejunum [19], supporting a mechanism related to the site of nutrient delivery rather than to the copper content of the feed.

The pathophysiological basis for this phenomenon relates to the sites of copper absorption. The intestinal copper transporter CTR1 is most highly expressed in the stomach and proximal duodenum, and gastric acidity facilitates the reduction of Cu^2+^ to Cu^+^, the form taken up by CTR1 [10,11]. Jejunal feeding bypasses both the acid-dependent solubilisation step and the highest-density absorption sites, resulting in reduced bioavailability even when nominal copper delivery is adequate. This mechanism may also explain the temporal pattern observed: deficiency emerged predominantly within the first six months of post-pyloric feeding, reflecting progressive depletion of hepatic copper stores once absorption is chronically impaired. Copper absorption is further modulated by its chemical form and by luminal factors such as dietary amino acids, protein source and ascorbic acid [29,30].

Insufficient copper content of the feeds is an unlikely explanation for our findings. All formulas used were standard, nutritionally complete products regulated as food for special medical purposes; those listed in Table 2 have a copper density of 72–100 μg/100 kcal, within the range required by EU legislation (60–500 μg/100 kcal) [7]. When prescribed at 1000 kcal/day, these products supply 2.1–2.9 times the IOM RDA for children aged 1–3 years and 1.6–2.3 times that for children aged 4–8 years, and 72–143% of the more generous EFSA AI [8,9]. Two caveats nevertheless apply. First, children with neurological impairment frequently have energy requirements well below those of healthy peers [3], and at reduced energy prescriptions the copper supplied falls proportionally, so that intake may approach or fall below the EFSA AI while remaining above the IOM RDA. Second, reference intakes assume physiological absorption through an intact stomach and duodenum and do not account for the reduced bioavailability that may follow direct jejunal delivery. In our cohort, the actual copper intake of the copper-deficient children was comparable to that of the JN patients who remained copper-sufficient (median 172% vs. 170% of the RDA; Section 3.1), and in several of them it was below the EFSA AI, indicating that the deficiency reflected impaired absorption rather than insufficient provision.

The clinical significance of copper deficiency in this cohort warrants emphasis. Two-thirds of affected patients developed overt haematological manifestations, predominantly anaemia and neutropenia. Copper plays an essential role in iron mobilisation through the ferroxidase activity of ceruloplasmin and contributes to normal maturation of myeloid precursors; deficiency may therefore mimic myelodysplastic syndrome or haematological malignancy and prompt extensive and invasive diagnostic workups [12,13,31,32]. The index case in this series illustrates this risk: bone marrow examination revealed trilineage dysplasia and ring sideroblasts before copper deficiency was identified as the unifying diagnosis. Awareness of this complication is essential to avoid unnecessary procedures in children receiving long-term post-pyloric feeding who develop unexplained cytopenias.

Iron deficiency was the most prevalent micronutrient abnormality in both groups, in keeping with previous reports [27], although this figure should be interpreted with caution because the operational definition relied on serum iron, which fluctuates diurnally and does not reflect iron stores; depleted stores, defined by ferritin or transferrin saturation, were confirmed in a smaller proportion of patients (Section 3.2) [23]. However, it appeared earlier and was numerically, though not significantly, more frequent in the JN group, suggesting that jejunal feeding may contribute independently to impaired non-haem iron absorption, or alternatively that the underlying disease severity was greater in this cohort. Selenium and zinc deficiencies were uncommon, asymptomatic, and mostly self-resolving, consistent with findings from other long-term EN cohorts [33].

A further mechanism deserves consideration when copper deficiency is found in a child on enteral nutrition. Excess zinc induces intestinal metallothionein, which sequesters copper and prevents its absorption, and is a recognised contributor to acquired copper deficiency [31,34]. In our cohort zinc intake was confined to that provided by standard paediatric formulas and no patient received zinc supplements, so this mechanism is unlikely to account for the findings; nevertheless, zinc intake should be reviewed whenever copper deficiency is identified.

Regarding treatment, all patients who received copper sulfate supplementation administered through the gastric route achieved full normalisation of serum copper and recovery of haematological parameters. This finding is relevant because previous case reports in adults described insufficient absorption of copper sulfate when administered directly through a jejunostomy tube, presumably due to the same bypass mechanism that causes deficiency [28]. Ensuring that copper supplementation is delivered via the gastric route—either through a separate gastric access or by temporary modification of the feeding route—appears to be critical for treatment efficacy.

The choice of copper salt deserves critical consideration. We used copper sulfate, an inorganic, highly water-soluble salt that dissociates readily in the acidic gastric lumen and serves as the reference compound in most bioavailability studies; by contrast, cupric oxide is poorly absorbed and should not be used for supplementation [35]. Amino acid chelates such as copper bisglycinate have been proposed as alternatives on the grounds that the chelated metal is protected from luminal antagonists and might be taken up through amino acid or peptide transport pathways present along the small intestine, which could theoretically be advantageous when the stomach and duodenum are bypassed [29,30]. This rationale, however, remains largely theoretical. Comparative data come almost exclusively from animal studies and are inconsistent; in cattle fed a diet rich in copper antagonists, for example, the relative bioavailability of copper bisglycinate was 82% of that of copper sulfate based on liver copper [36]. To our knowledge, no study has compared chelated and inorganic copper in humans fed post-pylorically, and at least some of the formulas used in our cohort already provide copper as an organic salt (copper gluconate), which did not prevent deficiency. The reported failure of copper sulfate delivered directly into the jejunum [28] suggests that the site of administration matters more than the chemical form. In the absence of comparative data, we would therefore prioritise gastric administration of a soluble copper salt and reserve intravenous copper, at the dose recommended for paediatric parenteral nutrition [37], for patients without gastric access or with persistent deficiency; whether a chelated preparation given through the jejunal port is an effective alternative remains an open question that merits prospective evaluation.

The potential utility of cocoa powder as a vehicle for copper supplementation, as reported by Nishiwaki et al. and others in adult cohorts [14,38], was not explored in this study. Paediatric dosing data for cocoa-based supplementation remain limited, and the tolerability and palatability of cocoa powder in young children, particularly those with altered gastrointestinal anatomy, would require specific evaluation. Copper sulfate solution administered through the gastric port proved effective and well-tolerated in all treated patients.

Based on the temporal pattern observed, we propose that micronutrient monitoring—including serum copper, iron, zinc, and selenium—should be initiated at three to six months after commencement of post-pyloric feeding and performed periodically thereafter, aligned with current ESPEN and ESPGHAN recommendations for long-term EN monitoring [1,4,5,22,39]. In patients with complex anatomy or established short bowel syndrome, earlier and more frequent monitoring may be warranted.

This study has several limitations. The single-centre design and relatively small sample size, particularly in the GN group, limit statistical power and the generalization of the findings. Although the difference in copper deficiency prevalence between groups was clinically striking (29.0% vs. 0%) and statistically significant (*p* = 0.021), the 95% CI of the risk difference was wide (4.9–46.6%), the analysis rests on only nine events, and the absence of events in the GN group precluded estimation of a relative effect measure. As in any observational study, the feeding route was not randomly assigned but dictated by clinical indication, and the JN and GN groups may differ in underlying disease severity, gastrointestinal anatomy (including previous surgery), formula type, duration of EN and use of acid-suppressive therapy (Table 1), all of which could influence micronutrient status independently of the feeding route. With nine events, none of them in the GN group, multivariable adjustment was not feasible and residual confounding by indication cannot be excluded; the association reported should therefore not be interpreted as causal. Reassuringly, the estimate was stable in sensitivity analyses excluding the oldest patient and the patient with short bowel anatomy. Determinations were also more frequent in the JN group (median interval 113 vs. 134 days; Table 1), which may have favoured earlier or more frequent detection of deficiency in that group (surveillance bias). Biochemical markers of copper status may be influenced by inflammatory status, as copper is a positive acute-phase reactant; however, CRP was measured in the same sample as every micronutrient determination and was below 2 mg/dL in all determinations used to classify micronutrient status (Table 4), so that an ongoing acute-phase response can be excluded as a confounder. Had inflammation been present it would have raised serum copper, leading to underestimation rather than overestimation of the prevalence of deficiency. A further methodological caveat is that micronutrient status was assessed on plasma concentrations alone, and paediatric reference thresholds for these trace elements are not fully standardised across laboratories and age ranges. Iron deficiency was defined on serum iron, an imperfect marker of iron status, and ferritin and transferrin saturation were available only for a subset of patients. Most importantly, the design was ambispective rather than strictly prospective. In 29 of the 46 participants the enteral device had been placed before the start of the recruitment period, and for these children the baseline panel and the determinations performed between initiation of EN and enrolment were retrieved from the clinical record; precise formula volumes for that earlier interval were likewise reconstructed retrospectively. In this subset the onset of deficiency could only be established as the date of the first determination below the reference limit, so the intervals reported represent upper-bound estimates conditioned by the sampling schedule rather than by a fixed protocol, and 2 of the 9 copper-deficient children could not contribute to that analysis at all. We have therefore refrained from describing the cohort as fully prospective. A multicentre study enrolling children at the time of enteral device placement, with a standardised baseline panel and predefined monitoring intervals, would be needed to confirm these findings and to develop evidence-based clinical guidelines.

## 5. Conclusions

In this single-centre cohort, prolonged jejunal enteral nutrition was associated with a higher prevalence of copper deficiency in paediatric patients compared with gastric feeding, even when standard enteral formulas provided copper intakes that met current reference values. Given the small sample, the wide confidence interval of the estimate and possible confounding by indication, this association should be confirmed in larger prospective multicentre studies. In our cohort, deficiency tended to develop within the first six months of post-pyloric feeding and frequently caused clinically significant haematological manifestations that were fully reversible with adequate supplementation. Pending such studies, we suggest that systematic surveillance of serum copper—and other micronutrients—be incorporated into standard monitoring protocols for all children receiving long-term jejunal enteral nutrition in order to prevent severe but avoidable complications.

## Figures and Tables

**Figure 1:**
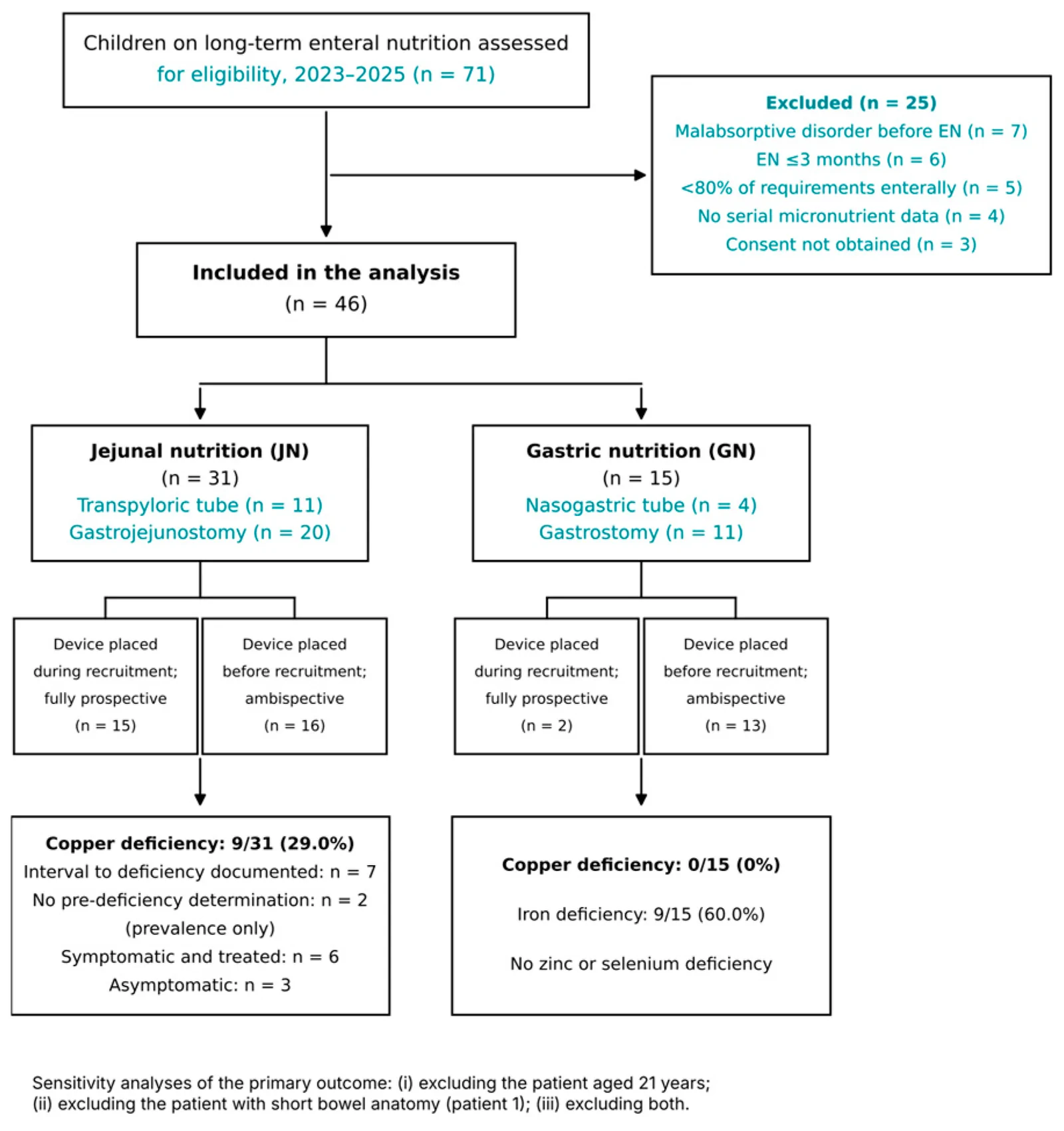
Flow diagram of patient selection, prepared according to the STROBE statement [21]. JN = jejunal nutrition; GN = gastric nutrition. “Fully prospective” denotes children enrolled at device placement; “ambispective” denotes children already on EN at the start of recruitment, whose determinations before enrolment were retrieved from the clinical record.

**Table 1:** Demographic and clinical characteristics of the study population.

Characteristic	Jejunal Nutrition (*n* = 31)	Gastric Nutrition (*n* = 15)	*p* Value
Male sex, *n* (%)	18 (58.1)	9 (60.0)	1.00
Age at enrolment, years, median (range)	7.0 (1.6–17.3)	6.0 (1.3–21.0)	0.91
Age < 10 years, *n* (%)	22 (71.0)	11 (73.3)	1.00
Enteral access device, *n* (%)			
Transpyloric (nasojejunal) tube	11 (35.5)	—	
Gastrojejunostomy	20 (64.5)	—	
Nasogastric tube	—	4 (26.7)	
Gastrostomy	—	11 (73.3)	
Clinical indication for the current feeding route, *n* (%)			
Dysphagia with gastro-oesophageal reflux	17 (54.8)	5 (33.3)	0.22
Severe GERD with recurrent aspiration pneumonia	5 (16.1)	0 (0)	0.16
Gastroparesis or other gastric dysmotility	4 (12.9)	0 (0)	0.29
Gastric outlet obstruction or anatomical cause	2 (6.5)	0 (0)	1.00
Intolerance of gastric feeding (vomiting, retching)	2 (6.5)	0 (0)	1.00
Insufficient oral intake or faltering growth without GERD	1 (3.2)	9 (60.0)	<0.001
Other (unsafe swallow after airway surgery)	0 (0)	1 (6.7)	0.33
Underlying diagnosis, *n* (%)			
Neurological impairment	11 (35.5)	6 (40.0)	1.00
Structural gastrointestinal anomaly (e.g., oesophageal atresia)	6 (19.4)	1 (6.7)	0.40
Genetic or congenital syndrome	6 (19.4)	3 (20.0)	1.00
Congenital heart disease	3 (9.7)	2 (13.3)	1.00
Mitochondrial, metabolic or neuromuscular disease	4 (12.9)	2 (13.3)	1.00
Other	1 (3.2)	1 (6.7)	1.00
Previous gastrointestinal surgery, *n* (%)	12 (38.7)	3 (20.0)	0.32
Short bowel anatomy, *n* (%)	1 (3.2)	0 (0)	1.00
Acid-suppressive therapy, *n* (%)	18 (58.1)	7 (46.7)	0.54
Formula type: polymeric/peptide/amino acid, *n*	19/12/0	12/3/0	0.32 †
Duration of EN through current route, months, median (range)	22 (4–37)	25 (6–59)	0.26
Interpretable copper determinations per patient, median (range)	5 (3–9)	4 (2–7)	0.11
Interval between determinations, days, median (range)	113 (55–158)	134 (99–224)	0.001
Formula copper content, μg/100 mL, median (range)	80 (75–120)	80 (75–120)	0.77
Daily copper intake, μg/day, median (range)	864 (499–1830)	888 (548–1784)	0.51
Daily copper intake, μg/kg/day, median (range)	46.5 (26.4–66.8)	49.3 (26.8–73.1)	0.35
Copper intake, % of IOM RDA, median (range)	171 (97–264)	200 (133–300)	0.17
Copper intake, % of EFSA AI, median (range)	86 (59–141)	96 (62–132)	0.38

GERD = gastro-oesophageal reflux disease; EN = enteral nutrition; RDA = recommended dietary allowance (IOM) [8]; AI = adequate intake (EFSA) [9]. Categorical variables were compared with Fisher’s exact test and continuous variables with the Mann–Whitney U test. † Polymeric vs. non-polymeric formula.

**Table 2:** Standard commercial paediatric enteral formulas used in the cohort: copper content and benchmarking against reference copper intakes.

Formula (Manufacturer)	Type	Intended Age	Energy (kcal/100 mL)	Cu (μg/100 mL)	Cu (μg/100 kcal)	Cu per 1000 kcal (μg)	% IOM RDA 1–3 y (340 μg)	% IOM RDA 4–8 y (440 μg)	% EFSA AI 1–<3 y (700 μg)	% EFSA AI 3–<10 y (1000 μg)
Infatrini Peptisorb (Nutricia)	Peptide-base, energy-dense infant	0–18 mo	100	75	75	750	221	170	107	75
Peptamen Junior PHGG (Nestlé)	Peptide-based	1–10 y	100	80	80	800	235	182	114	80
Frebini Original (Fresenius Kabi)	Polymeric	1–12 y	100	100	100	1000	294	227	143	100
PediaSure (Abbott)	Polymeric	1–13 y	100	100	100	1000	294	227	143	100
Nutrini Peptisorb Energy (Nutricia)	Peptide-based, energy-dense	≥1 y	152	110	72	724	213	164	103	72
Nutrini Energy Multi Fibre (Nutricia)	Polymeric, energy-dense, fibre	1–6 y	151	120	79	795	234	181	114	79

Cu = copper; RDA = recommended dietary allowance of the US Institute of Medicine (IOM) [8]; AI = adequate intake of the European Food Safety Authority (EFSA) [9]. Composition per 100 mL is taken from the manufacturers’ product data sheets. Percentages express the copper supplied by 1000 kcal of formula (approximately the daily energy requirement of a healthy child aged 1–3 years) relative to each reference value; in children receiving less energy, as is common in neurological impairment [3], copper supply decreases proportionally. For infants aged 7–12 months the reference values are 220 μg/day (IOM AI) and 400 μg/day (EFSA AI, 7–11 months). All products comply with the copper range required for nutritionally complete food for special medical purposes in the EU (60–500 μg/100 kcal; 60–120 μg/100 kcal for products for infants) [7].

**Table 3:** Prevalence of micronutrient deficiencies and median time to first deficit according to feeding route.

Micronutrient	JN, *n* (%)	GN, *n* (%)	*p* Value	Median Days to Deficit—JN	Median Days to Deficit—GN
Copper	9/31 (29.0)	0/15 (0)	0.021	170	—
Iron	22/31 (71.0)	9/15 (60.0)	0.51	194	264
Selenium	4/31 (12.9)	0/15 (0)	0.29	—	—
Zinc	1/31 (3.2)	0/15 (0)	1.00	—	—

JN = jejunal nutrition; GN = gastric nutrition. *p* values from two-sided Fisher’s exact test. Copper: risk difference 29.0% (95% CI 4.9–46.6%; Newcombe hybrid score method [24]). Median days to deficit are calculated among patients with a documented interval (copper, 7 of 9 patients) and represent upper-bound estimates, because onset was dated to the first deficient determination. Iron deficiency was defined by serum iron < 65 μg/dL (see Section 2.4).

**Table 4:** Serum concentrations of micronutrients and ceruloplasmin during follow-up according to feeding route.

Parameter	Reference Interval	Jejunal Nutrition (*n* = 31)	Gastric Nutrition (*n* = 15)	*p* Value
Copper (μg/dL)	70–150	89.2 (48.5–127.3)	98.9 (79.0–112.7)	0.03
Ceruloplasmin (mg/dL)	20–60	22.5 (12.7–30.6)	25.8 (19.4–30.5)	0.02
Iron (μg/dL)	65–175	55.4 (32.0–94.9)	56.9 (44.6–88.0)	0.34
Zinc (μg/dL)	45–110	82.9 (57.1–119.7)	93.3 (65.2–121.5)	0.07
Selenium (μg/L)	60–120	75.4 (45.1–96.3)	78.2 (63.9–96.1)	0.20
*C*-reactive protein (mg/dL)	<2 *	0.30 (0.07–0.75)	0.29 (0.13–0.73)	0.99

Values are expressed as median (range) of the per-patient summary values (each patient contributing the median of his or her interpretable determinations obtained during follow-up). Between-group comparisons were performed with the Mann–Whitney U test. * Threshold below which serum copper and ceruloplasmin are considered interpretable as nutritional markers, per the ESPEN micronutrient guideline [22]; all determinations included in this table met this criterion.

**Table 5:** Clinical and biochemical characteristics of patients with copper deficiency (*n* = 9), all belonging to the jejunal nutrition group.

Patient	Age (y)	Sex	Diagnosis	Access	Serum Cu (μg/dL)	Clinical Features	Treatment
1	2	F	Down syndrome + EA type 1 + SBS	GJ tube	6.9	Severe anaemia + neutropenia	Copper IV → oral Cu sulfate; normalised at 2 months
2	5	M	Neurological impairment	TP tube	18	Anaemia	Cu sulfate 40 μg/kg/day; normalised
3	3	F	Congenital heart disease + dysphagia	GJ tube	22	Anaemia + neutropenia	Cu sulfate 45 μg/kg/day; normalised
4	7	M	CHARGE syndrome	TP tube	31	Anaemia	Cu sulfate 40 μg/kg/day; normalised
5	4	F	Cerebral palsy + dysphagia	GJ tube	28	Neutropenia only	Cu sulfate 50 μg/kg/day; normalised
6	9	M	Esophageal atresia repair + dysphagia	TP tube	35	Anaemia + neutropenia	Cu sulfate 45 μg/kg/day; normalised
7	6	F	Mitochondrial disease	GJ tube	41	Asymptomatic	Spontaneous normalisation
8	11	M	Congenital myopathy	GJ tube	38	Asymptomatic	Transition to gastric feeding
9	3	F	VACTERL association	GJ tube	44	Asymptomatic	Spontaneous normalisation

EA = esophageal atresia; SBS = short bowel syndrome; Cu = copper; GJ = gastrojejunostomy; TP = transpyloric; IV = intravenous; F = female; M = male.

**Table 6:** Biochemical and haematological parameters of the nine patients with copper deficiency at the time of diagnosis and after supplementation.

Patient	Cu at Diagnosis (μg/dL)	Ceruloplasmin (mg/dL)	CRP (mg/dL)	Hb (g/dL)	Neutrophils (×10^9^/L)	Cu After Treatment (μg/dL)	Hb After Treatment (g/dL)
1	6.9	3	<2	6.0	0.62	94	11.4
2	18	9	<2	8.7	2.15	91	11.6
3	22	11	<2	9.2	0.85	96	11.8
4	31	15	<2	9.8	2.20	89	12.1
5	28	12	<2	11.7	0.72	92	12.0
6	35	16	<2	9.5	0.91	88	11.9
7	41	18	<2	12.2	2.60	86	12.3
8	38	22	<2	12.5	2.35	84	12.6
9	44	24	<2	11.9	2.10	82	12.1

Cu = copper; CRP = *C*-reactive protein; Hb = haemoglobin. Post-treatment values correspond to the first determination obtained after normalisation of serum copper (patients 7 and 9, spontaneous normalisation; patient 8, after transition to gastric feeding). CRP was below 2 mg/dL in every patient at the time of sampling, confirming the absence of ongoing systemic inflammation.

## Data Availability

The anonymised data supporting the findings of this study are available on request from the corresponding author, subject to approval by the Clinical Research Ethics Committee of Hospital Universitari Vall d’Hebron. The data are not publicly available due to privacy restrictions concerning paediatric clinical records.

## Abbreviations

The following abbreviations are used in this manuscript:

AI	Adequate intake
ASPEN	American Society for Parenteral and Enteral Nutrition
CI	Confidence interval
CRP	*C*-reactive protein
CTR1	Copper transporter 1
Cu	Copper
EA	Esophageal atresia
EFSA	European Food Safety Authority
EN	Enteral nutrition
ESPEN	European Society for Clinical Nutrition and Metabolism
ESPGHAN	European Society for Paediatric Gastroenterology, Hepatology and Nutrition
FSMP	Food for special medical purposes
GERD	Gastro-oesophageal reflux disease
GJ	Gastrojejunostomy
GN	Gastric nutrition
IOM	Institute of Medicine (United States)
IV	Intravenous
JN	Jejunal nutrition
PEG	Percutaneous endoscopic gastrostomy
PEJ	Percutaneous endoscopic jejunostomy
RDA	Recommended dietary allowance
SBS	Short bowel syndrome
STROBE	Strengthening the Reporting of Observational Studies in Epidemiology
TSAT	Transferrin saturation
